# Emergency management of incidental pulmonary embolism (IPE)

**DOI:** 10.1186/s44201-022-00004-7

**Published:** 2022-06-20

**Authors:** Carme Font, Tim Cooksley, Shin Ahn, Bernardo Rapoport, Carmen Escalante

**Affiliations:** 1 Department of Medical Oncology, Hospital Clinic Barcelona, Barcelona, Spain; 2 The Christie, Wilmslow Road, Manchester, M20 4BX UK; 3 Department of Emergency Medicine, Cancer Emergency Room, University of Ulsan College of Medicine, Asan Medical Center, Seoul, Republic of Korea; 4 The Medical Oncology Centre of Rosebank, Johannesburg, South Africa; 5 Division of Internal Medicine, The University of Texas MD Anderson Cancer Center, Houston, TX USA

**Keywords:** Incidental pulmonary embolism, Cancer, Anticoagulation, Ambulatory care

## Abstract

Venous thrombo-embolic (VTE) disease is a common cause of complications in patients with cancer and is the second most common cause of death in oncology patients other than the malignant disease. Whilst symptomatic VTE comprises the majority of such presentations to an emergency department (ED), incidental pulmonary embolism (IPE) is an increasingly frequent reason for attendance.

Many studies report that the consequences of IPE do not differ significantly from those with symptomatic presentations and thus most guidelines recommend using the same approach. The complexity of treatment in cancer patients due to increased prevalence of co-morbidities, higher risk of bleeding, abnormal platelet and renal function, greater risk of VTE recurrence, and medications with the risk of anticoagulant interaction are consistent across patients with symptomatic and IPE.

One of the initial challenges of the management of IPE is the design of a pathway that provides both patients and clinicians with a seamless journey from the radiological diagnosis of IPE to their initial clinical workup and management. Increased access to ambulatory care has successfully reduced ED utilisation and improved clinical outcomes in high-risk non-oncological populations, such as those with IPE.

In this clinical review, we consider IPE management, its workup, the conundrums it may present for emergency physicians and the need to consider emergency ambulatory care for this growing cohort of patients.

## Introduction

Venous thrombo-embolic (VTE) disease is a common cause of complications in patients with cancer and is the second most common cause of death in oncology patients other than the malignant disease [1, 2]. Symptoms potentially consistent with pulmonary embolism (PE) and deep vein thrombosis (DVT) are frequently encountered in cancer patients. Improved cancer outcomes, alongside therapies such as immune checkpoint inhibition that increase the risk of thrombotic complications [3, 4], means that the burden of thrombo-embolic disease in this cohort will continue to form a significant workload for those working in oncologic emergency medicine.

Co-ordination of radiology, oncology, and emergency medicine can be challenging and practice can vary significantly between healthcare settings. Whilst symptomatic VTE comprises the majority of such presentations to an emergency department (ED), incidental pulmonary embolism (IPE) is an increasingly frequent reason for attendance in the context of the growing use of CT scanning. In this clinical review, we consider the management of IPE, its workup, the conundrums it may present for emergency physicians, and the need to consider emergency ambulatory care for this cohort of patients.

## Definition/incidence

IPE is defined as an unsuspected filling defect in the pulmonary arteries identified on CT imaging performed for another indication, usually a routine staging scan to assess cancer disease status [5]. The patient is usually asymptomatic, although in some cases following detection, history taking will identify symptoms consistent with PE.

Multidetector CT scanners can provide good visualisation of the pulmonary arteries up to the subsegmental level and significantly increase detection of VTE [6]. The reported prevalence of IPE varies from 1.6 to 7.3% [7, 8]. In a recent systematic review, the median reported incidence of IPE was 3.36% with a wide range according to the underlying primary tumour [9].

## Clinical workup in the emergency department

In patients presenting to the ED with IPE, much of the traditional initial diagnostic workup could be perceived as being redundant and critical elements in the patient history must be documented. A proportion of patients will have some symptoms potentially attributable to PE and had chosen not to have them medically evaluated. Commonly mild overlapping symptoms may be potentially secondary to VTE and other concomitant conditions in the setting of patients with cancer (see Table 1).

**Table 1:** Common symptoms and underlying conditions in patients with cancer that may contribute to misdiagnose VTE

Chest symptoms	Shortness of breath Chest pain	Pulmonary embolism Pleural or pericardial effusion Superior vena cava syndrome
	Haemoptysis	Anaemia Infection Cancer-related asthenia Drug-related pneumonitis Radiotherapy lung toxicity
	Syncope Palpitations—tachycardia	Myocarditis Arrhythmia
Lower and/or upper limb symptoms	Oedema	Deep vein thrombosis Lymphoedema
	Pain	Lymphadenopathy Superior vena cava syndrome
	Cyanosis	Inferior vena cava syndrome Hypoalbuminaemia Arterial ischaemia

Assessing for symptoms of upper and lower limb DVT is essential. Establishing the current cancer stage, presence of cerebral metastases, previous VTE, previous significant bleeding or risk of bleeding, concurrent medications, and patient concerns regarding VTE development are necessary.

Clinical examination should focus on assessment of haemodynamic stability, respiratory compromise, and potential thrombotic sources. Exertional oxygen saturations should be measured [10]. Upper and lower limbs should be carefully examined for signs of DVT alongside exit sites of any indwelling catheters.

An electrocardiogram (ECG) should be performed as a minimum, but ideally a focused echocardiogram evaluating right ventricular function is completed [11]. Right ventricular function can be impaired due to increased afterload from a high-risk PE potentially leading to haemodynamic collapse. Point of care Doppler ultrasound could also be performed in the ED to assess for a peripheral DVT especially in patients with an isolated subsegmental PE [12].

In order to help determine the most appropriate anticoagulation strategy for IPE, blood counts assessing haemoglobin and platelet levels alongside renal and liver function need to be undertaken. Thrombocytopenia and impaired renal function are common in a cancer patient undergoing anti-cancer therapies and anticoagulation should be personalised if these are present. Troponin levels should be measured as a prognostic marker, and those with elevated levels should be treated as intermediate-high risk and considered for inpatient hospital observation and further management [13].

## Anticoagulation strategy in IPE

The complexity of treatment in cancer patients due to increased prevalence of co-morbidities, higher risk of bleeding, abnormal platelet and renal function, greater risk of VTE recurrence, and medications with the risk of anticoagulant interaction are consistent across patients with symptomatic and IPE. Many studies report that the consequences of IPE do not differ significantly from those with symptomatic presentations and thus most guidelines recommend using the same approach [14, 15]. Similar rates of recurrent VTE, major bleeding, and mortality have been reported in patients with symptomatic and IPE in several retrospective and observational studies [16–18]. However, a recent meta-analysis of 23 studies found that patients with IPE had lower rates of VTE recurrence at 6 months, with a trend towards higher incidence of major bleeding but no difference in mortality compared to those with symptomatic PE [19].

In a pooled analysis of 926 patients with IPE from 11 cohorts, at 6 months, the rate of recurrent VTE was 5.8%, major haemorrhage was 4.7% and mortality rate of 37% [20]. An international observational registry study of 695 IPE patients at 12 months reported these rates were 6%, 5.7% and 43% respectively [17]. The RIETE registry found that patients treated with anticoagulation for IPE had lower 90-day PE-related mortality than those with symptomatic presentations [21]. Proximal PE forms a significant proportion of IPE presentations with a reported prevalence of central IPE in the ED ranging from 23 to 65% [22].

The management of subsegmental PE in particular remains controversial. Although there is growing evidence that not anticoagulating patients with subsegmental PE is a safe approach, the data is mainly from patients without malignancy [23, 24]. One study of cancer patients reported that whilst symptomatic PE showed better survival with anticoagulation, anticoagulation did not result in significant survival benefit in IPE. Subgroup analysis showed significant improvement in survival with anticoagulation in proximal IPE but not in patients with distal IPE [25]. The current American Society of Clinical Oncology (ASCO) and American Society for Hematology (ASH) guidelines recommend treating incidental subsegmental pulmonary embolism on an individual case basis [26, 27].

Multiple landmark studies have demonstrated the non-inferiority of direct oral anticoagulants (DOACs) compared with LMWH in the management of cancer-related VTE. These include the SELECT-D trial [28], ADAM VTE study [29], and those performed by the Hokusai-VTE and Caravaggio investigators [30, 31].

Sub-analysis of the 331 patients with IPE in the Hokusai-VTE cancer study reported similar mortality rates to those with symptomatic PE, supporting current guidelines for the same management of the two presentations [32]. However, sub-analysis of the 232 patients of IPE in the Caravaggio study had lower rates of recurrent VTE but higher levels of major bleeding compared to those with symptomatic PE. Comparison of apixaban to dalteparin in the management of patients with symptomatic PE and IPE showed the hazard ratio for recurrence was 0.73 and 0.41, respectively, and for major bleeding 0.93 and 0.96, respectively [33].

In the ED, short-term treatment with low molecular weight heparin (LMWH) may be an appropriate strategy. This provides safe management of the IPE and enables long-term approaches to be determined subsequently by treating oncologists and haematologists who will have access to more clinical information and can determine a personalised management plan.

## Risk stratification in patients with IPE

Following the diagnosis of an acute PE, patients undergo assessment for the risk of complications and several scoring systems have been validated to identify low-risk patients who can be managed in an outpatient ambulatory setting [34, 35]. PESI (Pulmonary Embolism Severity Index) and simplified PESI, the two most validated clinical-physiological risk scoring systems, consider patients with cancer not to be low risk [37]. However, the outcomes related to PE in these patients are difficult to distinguish from the underlying malignancy. Therefore, several risk stratification models for cancer patients with PE have been developed but none is specifically focused on those with IPE (see Table 2) [36–40].

**Table 2:** Risk assessment models developed for cancer-related pulmonary embolism

POMPE-C [38]	RIETE [36]		EPIPHANY Index [40, 41]			Workup scenarios (4S rule) [39]			
Patient weight	Metastatic disease	+ 4	Clinical decision rule**	Presence ≥ 1 vs. none			**TA-UPE**	**SPE**	**UPE-S**
Respiratory rate (breath/min)^*^	Immobilisation	+ 2	ECOG performance status scale	≥ 2 vs. < 2		Setting at PE diagnosis	Outpatient	In/outpatient	In/outpatient
Oxygen saturation^$^	Age > 80 years	+ 1	PE-specific symptoms	Yes/no		PE suspicion	No	Yes	No
Heart rate > 100 bpm	Heart rate ≥ 110 bpm	+ 1	Pulse oximetry	SaO_2_ < 90% vs. ≥ 90%		Vital signs	Normal	Any	Any
Altered mental status^&^	Systolic BP < 100 mmHg	+ 1	Tumour response assessment***			Symptoms	No	Yes	Yes
Respiratory distress^Φ^	Body weight < 60 kg	+ 1	Surgery of the primary tumour	Yes/no					
Do not resuscitate status^¢^									
Unilateral limb swelling									
**Risk stratification**	**Risk class**	**30-day mortality**	**Tree modelling risk score°**	**15-day serious complications**	**Mortality**	**30-day mortality**			
30-day death probability according to math calculation	Class 1: low-risk < 2	0–4%	Low-risk	1.6%	0.3%		**TA-UPE**	**SPE**	**UPE-S**
	Class 2: intermediate-risk 2–4		Intermediate-risk	9.4%	6.1%		3%	21%	20%
	Class 3: high-risk 5–7		High-risk	30.6%	17.1%				
	Class 4: Very high-risk > 7	20–30%				No difference in MB or recurrence of VTE within 90 days of follow-up			

*BP* blood pressure, *bpm* beats per minute, *MB* major bleeding, *PE* pulmonary embolism, *VTE* venous thromboembolism, *TA-UPE* truly asymptomatic and unsuspected PE, *SPE* suspected PE, *UPE-S* unsuspected PE with symptoms

*Highest documented respiratory rate within previous 6 h

^**$**^Most recent pulse oximetry measured in room air

^**&**^Acute impairment in consciousness, new disorientation, delirium or confusion

^Φ^Dyspnea or increased work for breathing

^¢^Written or verbal desire of the patient not to be resuscitated

^**^Adaptation of Hestia’s exclusion criteria

^***^Progressive disease, unknown/not evaluated disease, complete or partial response, stable or no evidence of disease

°Within 15 days from PE

The EPIPHANY index, which was derived from a registry of symptomatic and IPE patients in 14 Spanish hospitals, stratifies patients into low, intermediate, or high risk of complications within 15 days of diagnosis [39, 40]. This index uses six variables (Hestia-like clinical decision rule, Eastern Cooperative Group (ECOG) performance scale, oxygen saturation, presence of PEspecific symptoms, tumour response, and primary tumour resection). It has been validated in an external study of 258 IPE patients presenting to EDs [41]. This index may be a useful adjunct in the risk stratification of cancer patients with IPE.

A study of IPE patients managed through the ED of the MD Anderson Cancer Center in Texas reported that in the absence of saddle PE, hypoxaemia and significant co-morbidities, these patients could be considered for ambulatory outpatient management with LMWH therapy [42]. A prognostic score incorporating performance status and the presence of new or worsening symptoms at the time of IPE diagnosis, with and without considering the presence of incurable malignancy, correlated with overall survival and early mortality in patients with IPE [43]. Analysis of a registry of 695 IPE patients found that respiratory symptoms within 14 days of the presentation and the ECOG performance status were the most consistent predictors of mortality [44].

## Pathways and ambulatory emergency management of IPE

**Fig. 1:**
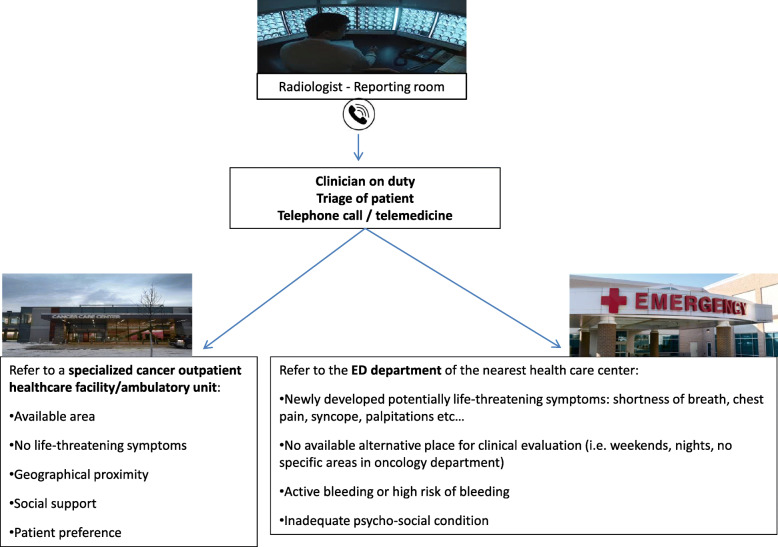
Proposed algorithm for the evaluation of patients with IPE

One of the initial challenges of the IPE management is the design of a pathway that provides both patients and clinicians with a seamless journey from the radiological diagnosis of IPE to their initial clinical workup and management. This will vary across acute care systems due to the heterogeneity of design but needs to be carefully considered and implemented in each setting (see Fig. 1).

Emergency care systems face a challenge of increasing demand on a backdrop of fixed resources for inpatient care [45]. Patients with cancer seeking emergency care generally have longer lengths of stay, higher admission rates, and higher mortality than non-cancer patients [46]. Ambulatory care is increasingly recognised as an essential component in the delivery of safe and sustainable emergency care. It aims to reduce the pressures and risks of ED overcrowding, which have been highlighted by the COVID-19 pandemic [47]. Increased access to ambulatory care has successfully reduced ED utilisation and improved clinical outcomes in high-risk non-oncological populations, such as those with PE [48].

The fundamental basis for ambulatory care is that patients presenting with acute illnesses can be stratified as low risk for developing complications and therefore do not require traditional inpatient care. Several models have been adapted to deliver this care including hospital at home, ambulatory care units, and observation units [45, 49].

Individualised management of emergency cancer presentations is a key challenge for oncologic emergency medicine. This requires collaboration and innovative development of models and services that facilitate this care. An increasing number of oncologic emergency medicine presentations, such as IPE, can be risk assessed for care in this setting [50, 51].

The distress of cancer-associated thrombosis can be significant and ameliorated by access to specialist services, information, and support [52]. Well-designed ambulatory emergency pathways for IPE will thus improve clinical outcomes, reduce pressure on overcrowded services, and help reduce the patient’s psychological burden due to IPE.
